# A Comparison of the Maximum Entropy Principle Across Biological Spatial Scales

**DOI:** 10.3390/e21101009

**Published:** 2019-10-16

**Authors:** Rodrigo Cofré, Rubén Herzog, Derek Corcoran, Fernando E. Rosas

**Affiliations:** 1 Centro de Investigación y Modelamiento de Fenómenos Aleatorios CIMFAV-Ingemat, Facultad de Ingeniería, Universidad de Valparaíso, Valparaíso 2340000, Chile; 2 Centro Interdisciplinario de Neurociencia de Valparaíso, Universidad de Valparaíso, Valparaíso 2340000, Chile; rubenherzog@ug.uchile.cl; 3 Departamento de Ecología, Facultad de Ciencias Biológicas, Pontificia Universidad Católica de Chile, Santiago 8331150, Chile; 4 Instituto de Ecología y Biodiversidad, Santiago 8331150, Chile; 5 Centre for Psychedelic Research, Department of Medicine, Imperial College London, London SW7 2DD, UK; f.rosas@imperial.ac.uk; 6 Data Science Institute, Imperial College London, London SW7 2AZ, UK; 7 Centre for Complexity Science and Department of Mathematics, Imperial College London, London SW7 2AZ, UK

**Keywords:** maximum entropy principle, biological systems across scales, model-free data analysis, inverse problems

## Abstract

Despite their differences, biological systems at different spatial scales tend to exhibit common organizational patterns. Unfortunately, these commonalities are often hard to grasp due to the highly specialized nature of modern science and the parcelled terminology employed by various scientific sub-disciplines. To explore these common organizational features, this paper provides a comparative study of diverse applications of the maximum entropy principle, which has found many uses at different biological spatial scales ranging from amino acids up to societies. By presenting these studies under a common approach and language, this paper aims to establish a unified view over these seemingly highly heterogeneous scenarios.

## 1. Introduction

While the scientific endeavor is traditionally associated with the divide et impera motto, the last decades have witnessed a shift in many areas of research towards considering the collective properties of sets of interacting elements such as cells, circuits of neurons, brains, species, and ecosystems [1]. This interest is fostered by the growing understanding that “more is different”, i.e., that many of these systems exhibit emergent properties that cannot be explained by the nature of their parts in isolation [2]. Another driver of this shift is the increasing amount of data available for analysis, which are enabled by novel recording techniques and recent advances in technologies for information storage, transfer, and analysis [3].

Biological research is, nowadays, in a peculiar situation: while there are more data available than ever before spanning all spatial biological scales, there is still a lack of an operational theory to explain the collective behavior of living organisms on different scales. One approach to pave the road towards finding such principles is to employ data-driven modeling techniques from the statistics literature, which—due to their generality—can be applied in diverse biological scenarios. While this way of proceeding might go against the traditional wisdom built by means of mechanistic considerations, a number of articles have shown that collective behavior can be effectively characterized by statistical models constructed purely from data [4,5], importantly, these studies show that is possible to build accurate statistical models without the need to characterize the mechanistic interactions or biological processes from first principles.

The maximum entropy principle (MEP) is one of the statistical methods that have found applications over a surprisingly wide range of biological scenarios. The core idea of the MEP is to build statistical models that agree with data, but are otherwise as “structureless” as possible. In other words, the MEP provides a method to find the least biased model that is consistent with the data, i.e., the maximally noncommittal with regard to missing information [6]. The initial success of the MEP method in physics and engineering rapidly triggered a plethora of applications in biology, including DNA motifs of transcription factor binding sites [7], co-variations in protein families and amino acid contact prediction [8,9,10], diversity of antibody repertoires in the immune system [11,12], collective activity of neural populations [4,5,13,14,15,16,17,18,19], collective behavior of bird flocks [20,21], collective behavior in groups of mice [22], and ecology of abundance and distribution [23,24]. The fact that the MEP has been successfully applied in these highly heterogeneous scenarios suggests that there might exist interesting organizational commonalities across biological scales. However, these commonalities are obscured by the parcelled language and terminologies employed in the various sub-disciplines of biology, which makes comparative studies highly non-trivial.

To help the exploration of common organizational properties, this article provides a unified review of recent advances related to the applications of the MEP across biological scales. The target audience for this article are researchers already familiar with the MEP, but are not aware of other applications in the realm of biology.

There are recent reviews related to the MEP applied to different fields of biology, with a focus on, e.g., parameter inference [25,26,27], reverse engineering [28], the learning of hidden variables [29], and information-processing in biophysical systems [30]. To complement this literature, the goal of this article is to provide a comparative study of applications of the MEP across biological spatial scales, providing a unified formalism, perspective, and notation that can bridge the differences between various scientific sub-fields. We compare and highlight some differences, extensions, and limitations of the MEP approach, and discuss open challenges for future research.

## 2. Maximum Entropy Principle: Preliminaries and Fundamentals

When studying living systems from data, scientist are usually unable to access all of the relevant information that would be required to fully characterize the system of interest. This limitation seems not to be a mere technological issue, but rather an intrinsic characteristic of biology—at least in the foreseeable future. For example, it is unlikely to be able to simultaneously measure the firing patterns of each of the $\approx 10^{11}$ neurons in the human brain, or quantify and classify all of the insects that live in the Amazonian rain-forest at a given time. Despite this limitation, it is usually possible to obtain accurate estimations of global properties from incomplete data, e.g., the average value of certain quantities of interest. Therefore, it is often relevant to find models consistent with this accurate—but partial—information.

An additional challenge is the fact that usually there are an infinite number of statistical models that are consistent with a given set of global properties measured from data. Therefore, one needs an additional criteria to decide which model to use. The MEP provides a rational basis to guide the model selection stage in these situations. The core of the MEP method is based on a constrained optimization problem of a concave functional—the Shannon entropy—resulting in a unique probability distribution that is consistent with the partial information at hand, being otherwise as “random” as possible.

In the rest of this section, we introduce the MEP from a broader perspective, i.e., as an inverse problem from statistical mechanics. After introducing the basic building blocks of this principle, we set the notation and fundamental ideas to unify the approach presented in the remaining sections.

### 2.1. Forward versus Inverse Problems

The idea of maximizing entropy has its origin in thermodynamics and statistical mechanics. In these branches of physics, there are two opposite approaches to build statistical models for characterizing the phenomena under study. One is to assume complete knowledge of the relevant mechanistic interactions that rule the constituents of the system, which is known as forward modeling. This approach provides a probabilistic model, which in turn determines values for the average of various observables of the system. The second approach works backward, hence it is called inverse modeling: one uses data to determine the average value of various observables, and then builds the “least structured” statistical model that is consistent with those values.

#### 2.1.1. Forward Modeling

The primary goal of statistical physics is to derive observable quantities from microscopic laws governing the parts and interactions of a system. The standard way in which these ideas are introduced is through Hamiltonian models that describe the interactions of a system from first principles. This approach is often called “forward,” and the goal is to characterize observables representing collective phenomena such as spin magnetization, correlations, or to characterize phase transitions as a function of the physical parameters of the model.

#### 2.1.2. Inverse Modeling

The inverse problem starts by taking average values of observables from data. The goal of this approach is to infer the parameters of a candidate Hamiltonian that defines the rules of local interactions, and in turn characterize the system using only data. Although some branches of biology have rich theories [31], there are other domains for which no mechanistic far-reaching accounts are available yet; in the latter cases, the MEP is usually among the best alternatives. Additionally, this approach can be used in any branch of biology that wants to “confirm” the parameters or interactions of a candidate Hamiltonian from data. Unfortunately, inverse problems are usually hard to solve; in particular, the application of the MEP to biological data usually relies on sophisticated numerical algorithms and computational power [4,16,17,32].

### 2.2. Maximum Entropy Principle: Definitions and Methods

The MEP is an inverse problem commonly employed in statistical mechanics, which has found applications in several other scenarios. This approach can reconstruct local rules of interaction from the data, without adopting specific mechanistic assumptions. While the MEP can take a very general form (see, e.g., [33]), this article focuses on the standard approach that focuses only on average values computed from data. In the next section, the basic building blocks of the MEP are explained, while introducing the notation that is used throughout the rest of the article.

#### 2.2.1. State Space, Observables, and Average Values

Suppose that scientists are interested in a particular system, for which they only have data but otherwise no additional knowledge. The first step in building the maximum entropy model of the system is to describe the set of possible configurations, which is called state space. For concreteness, let us consider a system made of *N* sub-units $x=(x^{1},\cdots ,x^{N})$, where each coordinate $x^{i}\in X^{i}$ represents the state of each sub-unit. The state space is denoted by $X=X^{1}\times X^{2}\cdots \times X^{N}$. It is important to note that the state space grows exponentially with the number of sub-units. Therefore, when considering systems composed by many sub-units, the state space is usually too large to be characterized directly from data, i.e., the cardinality of the state space is usually much larger than the number of data samples.

Having a clear idea of the state space of the system, the second step is to choose the observables of the system. Observables are real-valued functions on the state space of the form:

$$\begin{array}{cc} f:X & \to R\\ x & \mapsto f(x)\;. \end{array}$$

Observables are random variables whose average values can be estimated from data. With the state space defined and the estimation of basic statistical features with sufficient accuracy, the scene is set to build the minimally structured model that is consistent with these measurements.

#### 2.2.2. Entropy Maximization under Constraints

Although the concept of entropy was first used by Rudolf Clausius in the field of thermodynamics to study the relationship between energy and temperature, Shannon entropy [34] has a much broader scope dealing with the notions of information and uncertainty. Mathematically, for a discrete random variable with discrete probability distribution *q* over the state space X, its entropy is

(1) $$S[q]=-\sum_{x\in X}q\left(x\right)\log q\left(x\right).$$

The notion of entropy can also be applied to continuous variables via the differential entropy [35]. In this case, sums over the discrete state space are replaced by integrals:

(2) S[q]=−∫q(x)logq(x)dx

Shannon entropy can be understood as a generalization of the principle of insufficient reason, and Laplace’s principle of indifference. The basic formulation of these principles state that, if one is interested in assigning probabilities to events, but there is no further information available about them, one should assign the probabilities using the uniform distribution (which is the distribution that maximizes entropy when the number of events is finite). The MEP generalizes this to scenarios with infinite events, or when information about the system is available. For these cases, the MEP states that one should assign probabilities according to the distribution that maximizes the entropy while being consistent with the available information.

The inputs for the MEP are the average values of a set of observables, which represent the knowledge obtained from the data. As the empirical average of observables are usually not enough to uniquely determine a probability distribution, the MEP is used to obtain the unique probability measure *p* that maximizes the entropy among all the probability measures *q* that match the expected values of all of the observables. The MEP can be stated mathematically as the following optimization problem:

$$\begin{array}{cccc} \; & \max_{q\in M}\; & \; & S[q]\\ \; & \mathrm{subject}\;\mathrm{to}\; & \; & E_{q}[f_{k}]={\langle f_{k}\rangle }_{e},\;\forall k\in \left\{1,\cdots ,K\right\}, \end{array}$$

where M is the set of probability measures, $E_{q}\left[f_{k}\right]$ is the average of the observable $f_{k}$ for all k∈{1,⋯,K} with respect to *q*, and ${\langle f_{k}\rangle }_{e}$ is the empirical average of $f_{k}$ obtained from data. For more information about the technical aspects of the MEP, please refer to [6,17,18,36,37].

## 3. Examples at Different Spatial Scales

One of the most powerful features of the MEP is its generality, which enables its use over a extremely broad range of scenarios. This section explores six case studies of the application of the MEP in biology at different spatial scales, employing a unified methodology and notation. The cases are the following: amino acids in proteins (Section 3.1), retinal ganglion cells (Section 3.2), whole brain networks (Section 3.3), plant communities (Section 3.4), macroecologic biodiversity (Section 3.5), and human vote interactions in the US Supreme Court (Section 3.6). Rather than reviewing many articles related to each scale, we follow particular articles that summarize well the application of the MEP at that particular spatial scale. For each scenario, we describe the state space, the chosen observables, and the inferred information.

### 3.1. Amino Acid Interactions in Proteins

Proteins are sequences of amino acids, whose three-dimensional arrangement (tertiary structure) is critical for determining its biological function. An example of this is the case of homolog proteins: proteins with a common evolutionary ancestor, where the tertiary structure and biological function is highly conserved, while the amino acid sequence may differ in many ways. A family of homolog proteins can be summarized in the so-called “Multiple Sequence Alignment” (MSA): a matrix where all the sequences belonging to a given protein family are aligned using sophisticated similarity methods [38,39]. Large databases of MSA for different protein families are nowadays available (see, e.g., [40]) and the number will keep increasing as more complete genomes are sequenced [41].

This important technological advance faces the challenge of finding statistical regularities between the protein tertiary structure and the linear amino acid sequence. In fact, if the biological function of a protein highly depends on its tertiary structure, and the conservation of the tertiary structure depends on the conservation of interactions between amino acids residues, then it is expected that some sites of the amino acid sequences co-evolve, thus preserving its biological function.

Here, we review the results reported by Cocco et al. [42], focusing on showing explicitly the basic building blocks of the MEP, i.e., the state space, the observables, and the inferred information. In the article, the authors showed a wide variety of applications of MEP to the analysis of MSA as the prediction of co-evolving sites, physical interactions between amino acid residues on the tertiary structure, and the prediction of biological functions from the linear amino acid sequence.

#### 3.1.1. State Space

Consider a protein family composed of M homolog protein sequences (either from different species or different pathways inside the same specie). To align sequences of different lengths, a gap, “−”, may be introduced in some sequences. Therefore, there are r=21 possible values for each sequence site, namely 20 amino acids plus a gap, and hence we use $X^{i}=\left\{A,C,D,E,F,G,H,I,K,L,M,N,P,Q,R,S,T,V,W,Y,-\right\}$ (each letter represents one amino acid in the conventional representation).

**Figure 1:**
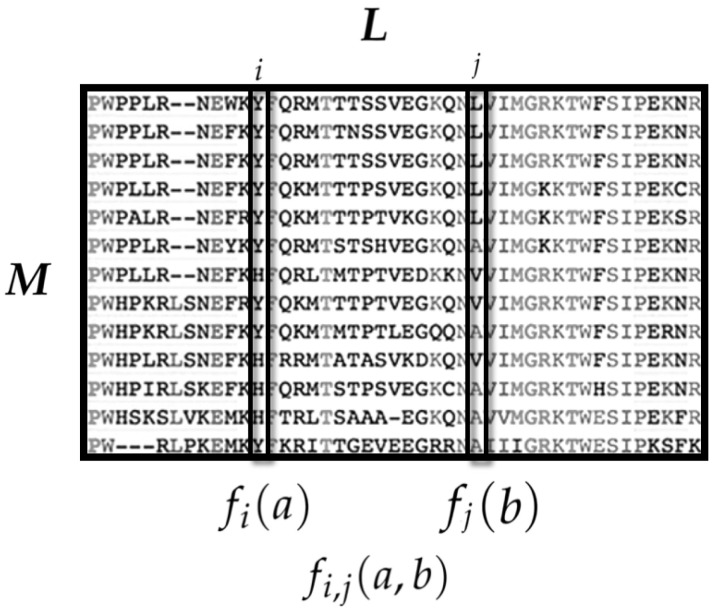
The amino acid sequences of *M* homolog proteins are aligned in the Multiple Sequence Alignment (MSA) matrix. Each MSA column is a sequence site and each row is the sequence of a member of the protein family. To have a fixed sequence length *L*, a gap (“−”) may be introduced. From the MSA, two sets of observables are considered: (i) $f_{i}\left(a\right)$ is the occurrence of the amino acid *a* at the site *i*; and (ii) $f_{ij}\left(a,b\right)$ is the co-occurrence of amino acid *a* at the site *i* and amino acid *b* at the site *j*.

Let us denote by *L* the aligned length of each protein sequence. Then, the MSA can be represented as a M×L matrix (see Figure 1), where the sequence of each protein is a *L*-vector and each sequence site *l* corresponds to one of the 21 amino acids from the $X^{i}$ set. However, for technical reasons, Cocco et al. [42] used a binary embedding, where each sequence is represented as a binary Lr vector, where the *i*th entry of each sub-vector is 1 if the corresponding *i*th amino acid of the $X^{i}$ set is present on that site and 0 otherwise. Thus, the state space is conformed by all the possible amino acid sequences of length *L*, i.e., $X={\left\{0,1\right\}}^{Lr}$.

#### 3.1.2. Observables and Average Values

The observables of interest are single site occurrence rates and pairwise occurrence rates. Hence, their average values consider both the single site averages and the pairwise site-correlations statistics:

- $\langle f_{i}\left(a\right)\rangle =\langle a^{i}\rangle$ is the average occurrence of the amino acid *a* at the *i*th sequence site.
- $\langle f_{i,j}\left(a,b\right)\rangle =\langle a^{i}b^{j}\rangle$ is the average co-occurrence of the amino acids *a* at the *i*th site and *b* in the *j*th site.

#### 3.1.3. Inferred Information

The statistical models built from the MEP yield valuable information about protein tertiary structure, its function, and protein design. Among the inferred information is the following.

1. **Co-evolving site pairs:** The interaction strength between site *i* and site *j* can be obtained as a function of the model parameters $J_{i,j}\left(a,b\right)$, i.e., the interaction between amino acid *a* in site *i* with the amino acid *b* in site *j*. This coupling strength can be used to identify evolutionary constraints on the site-interactions of the protein family.
2. **Contact Prediction:** The protein tertiary structure is associated with a topology of contacts between far amino acids residues. Interestingly, this topology can be inferred from the $J_{i,j}\left(a,b\right)$ coefficients. For predicting the tertiary structure of proteins, interactions between sites with a minimum separation of five sites on the linear sequence are usually studied—which is equivalent to one turn in an α-helix. The MEP approach outperforms the pairwise site contact prediction given by correlation-based methods (e.g., mutual information) [25,26,42], illustrating the power of considering interactions instead of mere correlations.
3. **Protein Design and the Effect of Mutations:** According to the energy landscape theory of protein folding [43], proteins conserved along evolution tend to minimize their free energy in their folded state. Using the MEP, the energy of each amino acid sequence can be computed, which allows to score each sequence according to its energy. This results in a set of non-naturally occurring proteins that minimizes the energy and, possibly, preserves the same functions as the original protein family. This inferred information has been applied to test and predict the effect of mutations [44,45].

To conclude, it is worth noting that the MEP approach represents an appealing alternative to computationally expensive molecular dynamics simulations.

### 3.2. Retina

The retina is a part of the brain which is located in the back part of the eye. Its main function is to encode different aspects of the visual stimulus and convey information to the visual brain areas about the visual stimulus, namely size, color, and movement, through the retinal ganglion cells as sequences action potentials (spikes) and silences. The integration and transformation of the stimulus from retinal ganglion cells constitutes the first stage of our visual perception. Multi-electrode arrays allow the recording spikes from thousands of retinal ganglion cells responding simultaneously to light stimuli. Since the seminal work of Schneidman et al. [13] and Pillow et al. [46] to characterize the spike train statistics of the vertebrate retina responding to natural stimuli, a succession of research efforts have helped to better understand some aspects of the so called ’“retinal code” (i.e., the input–output relationship). Since then, the MEP approach has become a standard tool to build statistical models in this field [4,13,46,47,48], and it is more and more clear that genuine collective behavior in the retinal network can be characterized using the MEP from relatively weak correlations among pairs of neurons (Ising model) [13].

Here, we translate the results reported by Tkačik et al. [4] into our standardized terminology. In this article, the authors built an accurate maximum entropy model that matches the firing rate of each retinal ganglion cell, their pairwise correlations, and the distribution of summed spikes in the network at each time bin.

#### 3.2.1. State Space

**Figure 2:**
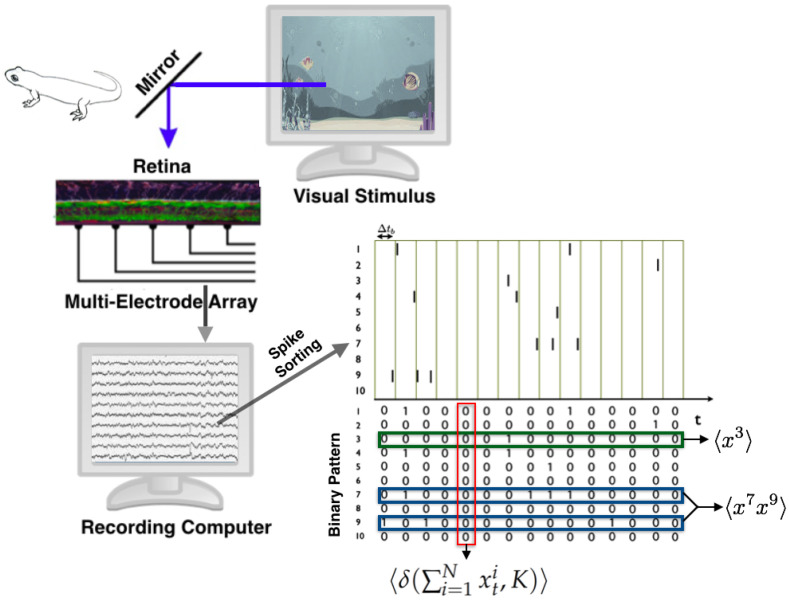
The retina of a vertebrate animal is extracted and mounted on the multi-electrode array in order to obtain the extracellular potential of the retinal ganglion cells responding simultaneously to natural stimuli. A signal processing procedure called spike sorting leads to the detection of the spikes of each cell. A binning procedure is applied to obtain binary patterns of activity, from which the average values of the observables are computed.

Consider a time discretization in which, for each time bin, each neuron can take only two values, either zero or one (see Figure 2). In the paper, the authors used a time window of $\Delta t_{b}=20$ ms. Consequently, in a network of *N* neurons, we denote $x_{t}^{k}$ the binary variable which takes value 1 whenever the *k*th neuron emits a spike during the *t*th time bin, and 0 otherwise. Therefore $X^{i}=\left\{0,1\right\}$ for all i=1,...,N.

This standard procedure transforms data into sequences of binary patterns (see Figure 2). A spike pattern is the spike-state of all the network at time bin *t*, denoted by $x_{t}:={\left[x_{t}^{n}\right]}_{n=1}^{N}$. Finally, a spike train or dataset is a finite sequence of spiking patterns. The state space is formed by all the spike patterns $X={\left\{0,1\right\}}^{N}$.

#### 3.2.2. Observables and Average Values

The observables and their average values used in this study are:

- $\langle f_{i}\left(x\right)\rangle =\langle x^{i}\rangle$: The firing rate of neuron *i*, for all neurons.
- $\langle f_{ij}\left(x\right)\rangle =\langle x^{i}\;x^{j}\rangle$: The synchronous pairwise correlation between neuron *i* and neuron *j*, for all pairs of neurons.
- $\langle f_{K}\left(x\right)\rangle =\langle \delta \left(\sum_{i=1}^{N}x_{t}^{i},K\right)\rangle$, for K=1,..,N.

Above, δ(·) represents the Kronecker delta, which takes value 1 when both arguments are equal, otherwise it is zero. Please note that in this scenario 〈·〉 corresponds to a temporal average taken over the data (see Figure 2).

#### 3.2.3. Inferred Information

The inferred maximum entropy distribution is used to investigate the principles underlying population coding, i.e., how groups of neurons orchestrate their spiking activity characterizing the neural code. In the article, the authors used the maximum entropy distribution p(x) to investigate the following issues:

1. **Joint Shannon entropy:** To characterize the size of the neural vocabulary, the effective number of configurations is reduced to $2^{S}$ (1). The entropy represents the ability of the system to explore these available states, and hence assesses the capacity of the neural population to represent visual information. In this case, a low entropy shown that the expected frequency of spike patterns are extremely inhomogeneous.
2. **Classification of activity patterns into meta-stable collective modes:** The energy landscape inferred from the maximum entropy method presents valleys, which resembles a “clustering of patterns” of neural activity, but obtained without a particular metric for similarity among patterns.
3. **Redundancy:** From the inferred joint distribution p(x), the authors computed the conditional marginal distributions $p(x^{i}=1|x_{\backslash i})$, where $x_{\backslash i}$ means all x except *i*. They showed that the state of individual neurons is highly predictable from the rest of the population, characterizing in this way the level of redundancy in the neural population. This property is suggested to allow error correction capabilities.

### 3.3. Resting State Networks in the Human Brain

As presented previously, the pairwise maximum entropy model (MEM) accurately describes firing patterns in the retinal ganglion cells [4,13], but additionally firing patterns and local field potentials (LFPs) in human cortical tissues in vitro [14] and large-scale firing patterns in the visual cortex of monkeys and cats in vivo [49,50]. These findings suggest the idea that the human brain activity patterns during rest may accurately be described by pairwise MEMs.

Here, we discuss results reported by Watanabe et al. [51] focusing on the detailed description of the state space and observables and inferred information. In this article, the authors studied spontaneous brain activity (in the absence of a task, but awake) using functional magnetic resonance imaging (fMRI) data. This technique has revealed that different brain regions interact with each other during rest, forming several resting-state networks (RSNs) [52]. The RSNs, including the default mode network (DMN) and the fronto-parietal network (FPN), are highly reproducible across different healthy individuals and are considered to underlie cognitive processes.

#### 3.3.1. State Space

**Figure 3:**
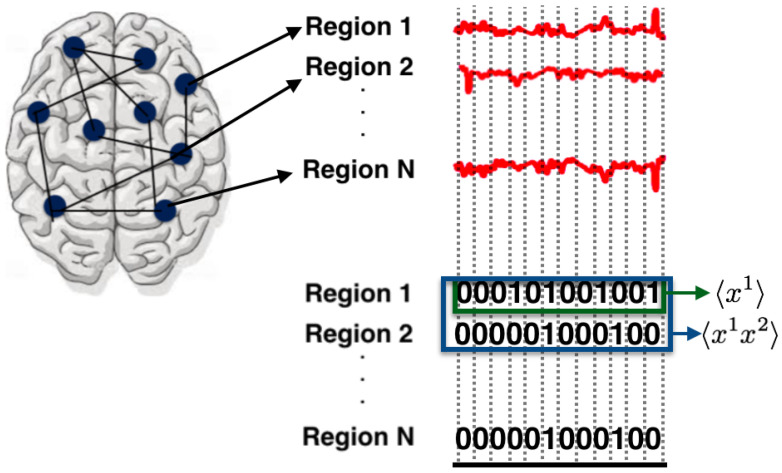
Regions of interest in the brain (represented as circles) corresponding to the DMN and FPN are selected and their BOLD signals (continuous in time and state-space) are analyzed. To obtain binary states, as in the previous example, the time is discretized choosing time windows of 9.045 s and the BOLD signals are binarized using a threshold under which the continuous signal is zero and otherwise is one (for details about the threshold and robustness of the choice, please refer to [51]). From the binary data, the average values with respect to time of the observables are computed. The maximum entropy principle is used to find the unique joint probability distribution that maximizes entropy, which is consistent with constraints computed from data.

Blood Oxygen Level-Dependent (BOLD) signals are extracted from anatomically defined regions. Their study focuses on 12 regions for the DMN and 11 regions for the FPN. The continuous signals from these regions was binarized, i.e., at each time step, the region is considered to be inactive (i.e., 0) if the BOLD signal is below a given threshold; otherwise, the region is considered active (i.e., 1, see Figure 3). The threshold is set to maximize the accuracy of fit of the maximum entropy distribution.

The authors considered two separate datasets, one for the DMN and one for the FPN. Each dataset is a sequence of patterns of zeros and ones. The state space as in the case of retinal ganglion cells is $X={\left\{0,1\right\}}^{N}$.

#### 3.3.2. Observables and Average Values

The observables and average values used in the article are (see Figure 3):

- $\langle f_{i}\left(x\right)\rangle =\langle x^{i}\rangle$: The activation rate of region *i*, 12 for the DMN and 11 for the FPN.
- $\langle f_{ij}\left(x\right)\rangle =\langle x^{i}\;x^{j}\rangle$: The synchronous pairwise correlation between region *i* and region *j*, for all pairs of regions of the DMN and FPN.

#### 3.3.3. Inferred Information

The authors showed that the pairwise MEM accurately characterizes the statistical behavior of discretized BOLD signals in the human RSNs. Functional interactions (coupling parameters) from the pairwise MEM were similar to the anatomical connections. The authors showed that the agreement between the estimated matrices of functional interactions and the anatomical connections is more accurate than other methods such as: (1) Pearson’s correlation coefficients; (2) inverse Gaussian model; (3) partial correlation; and (4) mutual information method. These findings suggest that the large-scale human brain networks of resting state can be accurately modeled by a synchronous pairwise model MEM.

### 3.4. Plant Communities Relative Abundances

For a long time, ecologists have tried to build models to estimate the relative abundances of species in a community [53]. Knowing this structure gives insights on the functionality of the community and allows us to infer information about ecological interactions such as competition [54].

Inferring the relative abundance of species has been attempted using different alternatives such as population biology approaches, and, lately, there have been more attempts using what is known as “the Unified Neutral Theory of Biodiversity and Biogeography,” or simply neutral theory [55,56].

Another approach using MEP was developed by Shipley et al. [57] to estimate the relative abundances of species. Shipley’s method was the first to predict macroecological patterns (specifically, the species abundance distribution) from maximum entropy models using functional traits as observables rather than species identities.

This model measures the mean value of functional traits of plant species in order to predict community assemblies even for a 3000 square kilometer area with a pool of over 500 species [58]. This approach brought a substantial improvement in the prediction of plant relative abundances, without considering explicitly in the model any biological or ecological processes [59]. In the following section, we discuss the approach used by Sonnier et al. [58], using our conventions and notation. We present these results focusing on the state space, observables and inferred information.

#### 3.4.1. State Space

Consider a community of plants in a landscape $A_{0}$ composed of a pool of $S_{0}$ species. Within that landscape, we could have any combination of proportions of each species $i\in S_{0}$ given by $x^{i}$. The state space is:

$$\begin{array}{c} X=\{\left(x^{1},x^{2},\cdots ,x^{S_{0}}\right)|x^{i}\in \left[0,1\right]\;\mathrm{and}\;\sum_{i=1}^{S_{0}}x^{i}=1\}. \end{array}$$

Please note that using an adequate definition of the species pool is a crucial step, as this determines the state space and hence critically influences the model.

#### 3.4.2. Observables

The observables are the functional traits of the pool of species that might be in the community. Functional traits are morpho-physio-phenological traits that impact the growth, reproduction and survival of the species [60]. They represent features such as diameter at breast height, whole plant height, leaf, area per leaf, dry mass, and seed mass, among others.

**Figure 4:**
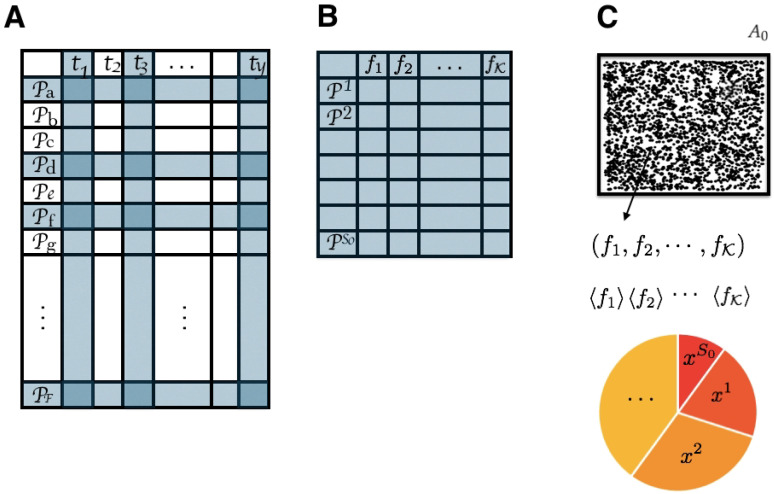
(**A**) From a large dataset where several plant species have their recorded mean trait value, we extract (**B**) a reduced database with the possible plant species present in $A_{0}$ (blue rows selected in (**A**)) and traits possible to be measured (blue columns selected in (**A**)). (**C**) Then, traits are measured in the field for all possible plants without specifying the species. The average values of these traits are the constraints for the maximum entropy problem of finding an estimate for the proportion of each plant species in $A_{0}$.

From a restricted area $A_{0}$, traits are measured from all of the plants found without identifying the plants. The average values of the observables (traits) are the constraints of the maximum entropy problem. The optimal proportion consistent with the observed data is found using the MEP and known databases where average traits are available for several plants (see Figure 4). Currently, in the Botanical Information and Ecology Network (BIEN) database, there are 53 traits that can be extracted for a large number of plant species, and if one of the traits is not available for the species level it can be inferred as the mean of the next available phylogenetic level (e.g., genus and family) [61]. There are also over 17 million plot observations where every plant has been recorded in a given area, thus there is a large database with more than 485,000 species with which to test this method.

#### 3.4.3. Inferred Information

The results of this model successfully predict the relative abundances of plant species throughout the landscape and over time [57]. The premise of Shipley’s model [57] is that the environment acts as a filter of species acting on functional traits, i.e., it is not the species identity but its traits that are important. As an example, this could lead to better predictions on how invasive a species could be in a new environment. Usually, you can predict whether a species can survive in a novel environment using species distribution modeling [62], but it is a lot more difficult to predict if that species will become abundant enough to be a problem. This model bridge the gap between community ecology and functional ecology.

### 3.5. Macroecology and Biodiversity

Macroecology is a field of ecology that studies ecosystems at a global scale looking for universal patterns and relationships. The objects of study are species–area relationship and species–abundance distributions, among others [63]. The MEP provides the theoretical framework to build data driven statistical models which can be used in this context to unify and study community patterns of macroecology through four variables which are measured in the field: number of species $S_{0}$, number of individuals $N_{0}$, the total area $A_{0}$, and total metabolic rate $E_{0}$. In most cases, the metabolic rate of individuals is inferred through scaling relationships, where individuals sizes predict their metabolic rate through known relationships [64].

Certainly, there are many ways to describe macroecology, using different ideas and mathematical techniques. There has been a recent effort to build unified theories of biodiversity [65].

Here, we present one version based in information theory, i.e., the Maximum Entropy Theory of Ecology (METE), which was introduced by Harte et al. [66,67], but called METE in the book [23], discussed in [68,69,70], and recently revisited to clarify some of the notation and incomplete derivations [71]. However, as discussed in [72], there is still work to be done in this theory. We use our notation and focus on the description of the state space and observables.

#### 3.5.1. State Space

One of the main problems faced by the METE is the estimation of the probability that a species picked at random in a chosen area $A_{0}$, belongs to a species that has a total population of *n* and with metabolic rate ϵ, given that in that area it is known that there is a pool of $S_{0}$ species, $N_{0}$ total individuals and a total metabolic rate of $E_{0}$. This probability is denoted by:

(3) $$p(n,\epsilon |A_{0},S_{0},N_{0},E_{0}),$$

where *p* is a mixed discrete distribution over *n* (discrete number of individuals) and continuous over ϵ (real valued metabolic rates). The state space is the product space between the abundance of each species and the total metabolic rate of each of them. Mathematically, in our notation, it is $X=N\times R_{+}$.

#### 3.5.2. Observables

The observables are the abundance per species *n*, whose average is estimated by the fraction $\frac{N_{0}}{S_{0}}$ where the numerator and the denominator are measured, and nϵ is the total metabolic rate of the individuals within the species, whose average over species is $\frac{E_{0}}{S_{0}}$. Please note that the values of these quantities can be extracted or estimated from existent databases. Both average values serve as the constraints of the MEP model:

(4) $$\sum {}_{n=1}^{N_{0}}\int_{\epsilon =1}^{E_{0}}n\cdot p\left(n,\epsilon |A_{0},S_{0},N_{0},E_{0}\right)\;d\epsilon =E_{p}\left[n\right]=\frac{N_{0}}{S_{0}}$$

(5) $$\sum {}_{n=1}^{N_{0}}\int_{\epsilon =1}^{E_{0}}n\cdot \epsilon \cdot p\left(n,\epsilon |A_{0},S_{0},N_{0},E_{0}\right)\;d\epsilon =E_{p}\left[n\epsilon \right]=\frac{E_{0}}{S_{0}}.$$

#### 3.5.3. Inferred Information

**Figure 5:**
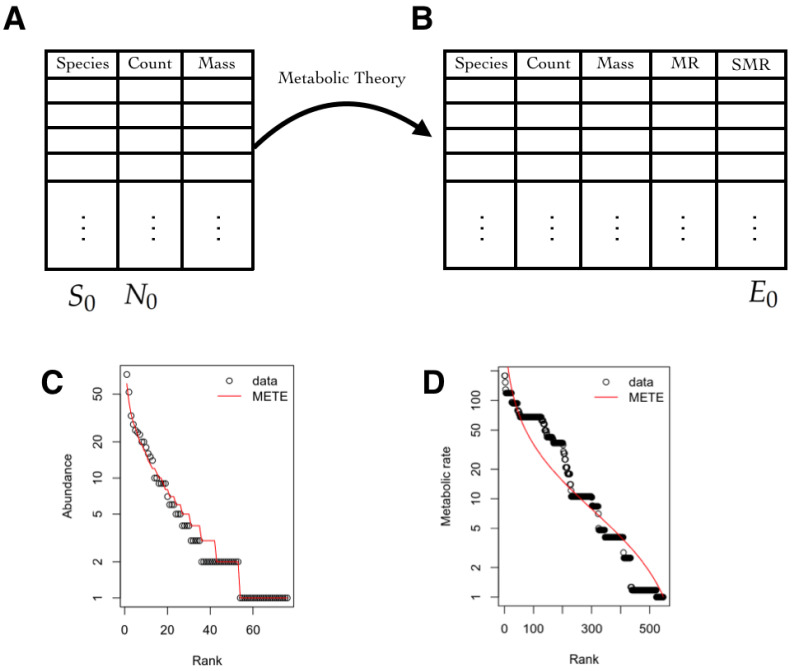
(**A**) Database with the species, their counts, and the mass of that species for a given area $A_{0}$. From here, the quantities used to compute the average value of the observables $S_{0}$ and $N_{0}$ are obtained; (**B**) Using the metabolic theory, the metabolic rate (MR) of each species is estimated. The quantity $E_{0}$ is computed from the standardized metabolic rate (SMR), which is obtained by dividing all of the MRs by the minimum MR; (**C**) The species-abundance distribution $\phi (n_{0}|A_{0},S_{0},N_{0})$ is computed from the joint maximum entropy distribution and a graph of rank versus abundance is plotted; (**D**) The metabolic rate distribution over all individuals is obtained $\psi (\epsilon |A_{0},S_{0},N_{0},E_{0})$. A graph of rank versus metabolic rate is shown. (**C**,**D**) Images were obtained from the maximum entropy distributions fitted to data available in the R package meteR [75] using Dan Gruner’s data [76].

Once the joint probability distribution *p* (3) is fitted by the MEP, many ecological relationships can be derived. For instance, one can obtain the marginal distributions. Integrating over ϵ, the species-abundance distribution is obtained which is usually denoted by $\phi (n_{0}|A_{0},S_{0},N_{0})$. Summing over *n*, the metabolic rate distribution over all individuals is obtained usually denoted by $\psi (\epsilon |A_{0},S_{0},N_{0},E_{0})$ (see Figure 5). Similarly, the species–area relationship and the endemics–area relationship, among other key features in macroecology, can be derived [24]. Recently, this methodology has been used to estimate *p* using data from 60 different forest communities, with more than 2000 species and it was proven to successfully explain both the species–area relationship and the individual–size distribution [73,74].

### 3.6. Human Voting Interactions in the US Supreme Court

Human interacting systems may show interesting collective behavior [77,78], which can be exhibited from data and from models that hypothesize the way in which they interact. In [79] the authors considered the voting of the Supreme Court of the United States (SCOTUS), which is the highest court in the US government. The article shows that the structure of coalitions among multiple justices can be studied using a pairwise maximum entropy model. Moreover, it is shown that a maximum entropy distribution provides surprisingly accurate descriptions of collective behavior in voting patterns.

#### 3.6.1. State Space

The data consist of N=9 justices who vote on the constitutionality of legislative and executive actions. The article considers data from the second Rehnquist Court (1994–2004, 895 votes) during a period in which the membership stayed constant. The Court issues majority and minority opinions, and these can be supplemented with other opinions; although opinions can be nuanced, each justice casts a yes $\left(x^{i}=+1\right)$ or no $\left(x^{i}=-1\right)$ vote, and the majority of votes decides the fate of each case. The state of the whole system can be represented by $x=(x^{1},\cdots ,x^{9})$. The state space of each variable is $X^{i}=\left\{-1,1\right\}$ and the state space of the whole system is $X=X^{1}\times X^{2}\times \cdots \times X^{9}$.

#### 3.6.2. Observables

**Figure 6:**
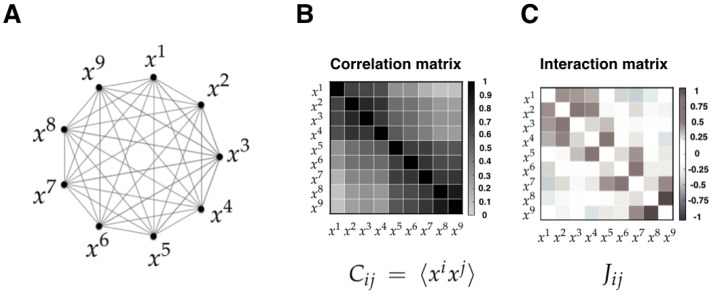
(**A**) Interacting random variables $x^{i}$ representing the votes of the nine justices; (**B**) Correlation matrix between random variables $x^{i}$ and $x^{j}$ measured directly from data; (**C**) Interaction matrix computed from the maximum entropy principle.

The model only considers pairwise correlations between the nine variables $C_{ij}=\langle x^{i}x^{j}\rangle$ (see Figure 6). These correlations are the restrictions of the maximum entropy problem. The maximum entropy distribution that is consistent with the observed pairwise correlations among justices’ votes is equivalent to an Ising spin glass with energy function:

$$H\left(x\right)=-\frac{1}{2}\sum_{i\neq j}J_{ij}x^{i}x^{j},$$

#### 3.6.3. Inferred Information

The MEP predicts the joint distribution over voting patterns p(x) and can be tested in various ways. For example, the probability that the vote is split (k,9−k), with k=[5,9] votes for the majority, can be computed from data and predicted from the model. Additionally, probability versus energy and mutual information are measured from data and compared with the predictions of the model. The article reports small quantitative discrepancies between the model and the data.

The MEM shows that voting patterns are organized in an energy landscape that is equivalent to an Ising spin glass. The authors of [79] insisted that this is not a metaphor, but a mathematical equivalence. This simple model correctly predicts the extent to which each justice is correlated with the majority without accounting for “ideologies”. A useful application of this MEM is that allows computing and ranking the influence that individual justices have on the majority decision. It can be observed that a strong tendency toward unanimity emerges from the inferred probability model.

This investigation shows that the competition between unanimity and ideological division emerges from interactions among the justices which can be inferred using the MEP under simple constraints even in a complex political context.

## 4. Discussion

In the previous sections, we outline the fundamental ideas behind the MEP and explore how it can be used to analyze data across different biological scales, ranging from the amino-acids up to macroscopic social scenarios (see Table 1). A key feature shared by all of these scenarios is the underlying randomness present in biological phenomena, which makes the probabilistic approach appropriate. Another important commonality is the impossibility of obtaining data that can cover the whole state space, which turns modeling and statistical inference into a necessity.

### 4.1. Lessons from the Case Studies

Let us emphasize some key takeaway messages obtained from the comparative studies presented in Section 3.

(i) **The MEP can be applied to a wide range of systems.** The flexibility of the MEP allows its application to biological systems. Moreover, the range of application not only spans spatial scales, but also includes technically diverse scenarios. In effect, in some of the considered case studies, the observables are directly related to causal/mechanistic interactions, while in others they are not. Moreover, the averages of these observables are in some cases temporal, while in other cases spatial. The fact that the same formalism can be adapted to such different contexts highlights the flexibility of the MEP approach [19].
(ii) **It is critical (and highly non-trivial) to choose an appropriate state space and observables.** While different applications of the MEP do not require conceptual changes to the basic method, the results rely entirely on the chosen state variables and observables, which are both determined by the modeler. For this reason, the researcher needs to double-check that these choices are adequate, i.e., if the model is capable of predicting (with some degree of accuracy) average values of observables not included in the MEP to fit the data, and if the model is capable of addressing the questions that one want to ask. It is crucial not to lose perspective on this issue; as the MEP is based on a concave maximization problem, it will always finds a unique solution, which might be useless if the state space and observables are not chosen appropriately.
(iii) **Correlations versus interactions**. It is important to note that the MEP makes a strong distinction between interactions and correlations. Indeed, correlations are statistical dependencies between variables, while interactions are the local rules of the system from which correlations and collective phenomena emerge. Importantly, each interaction term depends on all the correlation terms, and hence there exists no simple mapping between the correlation and the interaction between two sub-units. Furthermore, it has been shown that the interactions inferred using the MEP give a more useful account of the physical topology of some system than correlations. Examples of this include neural structural connectivity [51,80], and contacts between proteins sites [42]. In these examples, the inferred interactions obtained from the MEM parameters outperform linear or nonlinear correlation when predicting physical interactions between system variables. We believe that the key advantage of interactions over correlations is the fact that they faithfully reflect conditions of conditional independency, which are key in many statistical causal frameworks [81]. This crucial property might be behind the success of MEM in assessing emerging behavior in networks of interacting agents in biology [4,21,82,83,84].

### 4.2. Concluding Remarks

The MEP fits well the needs of biology in the era of big data, where information abounds but general principles—and corresponding mechanistic rules—are often scarce. Interestingly, what the MEP provides is remarkably different to other big data approaches such as deep learning, which usually focus in attaining accurate predictions without enabling insights about the underlying structure of the system. In contrast, the MEP often offers significant information about the system that goes beyond mere predictions, including statements about the physical interactions between its parts (e.g., Section 3.3), redundancy and metastability (e.g., Section 3.2), and insights on the structure of collective voting patterns (e.g., Section 3.6). Remarkably, all this added value is provided without the need of embracing a particular mechanistic description.

One of the main advantages of the MEP is that it is data driven. Therefore, the MEP can be used to test existing theoretical frameworks and generative mechanistic models existing in biology at different scales based on the data that these mechanistic models produce.

We expect the MEP approach to gather momentum as big data becomes ubiquitous at all scales of experimental biology. In effect, the MEP has the ability to support the analyses of the ever-increasing data, and pave the road towards new models of living systems based on their interactions, or towards confirming existing ones. Furthermore, the steady progress of technology is likely to open up new fields of application for inverse statistical analysis, in which the MEP might find novel fruitful applications. We hope that this article will contribute to the development of a broader understanding of the MEP across multiple biological scales, which in turn might help to foster new research avenues in the future.

## Figures and Tables

**Table 1:** Table of examples including the state space and the observables from which average values are calculated from data.

Scenario	State Space	Observables and Average values
Amino acids in proteins	${\left\{0,1\right\}}^{Lr}$	Average amino acid occurrence on a given site
		and average co-occurrences of amino acids on site-pairs
Retinal ganglion cells	${\left\{0,1\right\}}^{N}$	Firing rates and pairwise correlations
Whole brain networks	${\left\{0,1\right\}}^{N}$	Activation rates and pairwise correlations
Plant communities	${\left[0,1\right]}^{S_{0}}$	Average value of traits
Macroecologic biodiversity	$N\times R^{+}$	Average abundance per species and average over species
		of the total metabolic rate of the individuals within the species.
US Supreme Court	${\left\{-1,1\right\}}^{9}$	Pairwise correlations

## Abbreviations

The following abbreviations are used in this manuscript:

MEP	Maximum entropy principle
MEM	Maximum entropy model
DNA	Deoxyribonucleic Acid
MSA	Multiple sequence alignment
LFP	Local field potential
fMRI	Functional magnetic resonance imaging
BOLD	Blood Oxygen Level-Dependent signals
RSN	Resting state network
DMN	Default mode network
FPN	Fronto-parietal network
METE	Maxent Theory of Ecology
SCOTUS	Supreme Court of the United States
BIEN	Botanical Information and Ecology Network
**Symbol List**	
$x_{t}^{k}$	State of *k*th variable at time *t*
$x^{i}$	State of *i*th variable on **x**
x	Configuration of the N-elements of a system
$X^{i}$	State space of a random variable $x^{i}$
X	State space of a N-elements system
R	Set of real numbers
q[x]	Set of probability distributions that match ${\langle f_{k}\rangle }_{e}$
p[x]	Probability distribution that maximizes entropy and match ${\langle f_{k}\rangle }_{e}$
Sp	Entropy of the probability measure *p*
K	Number of observables
$f_{k}$	Observable *k*
${\langle f_{k}\rangle }_{e}$	Empirical average value of observable *k*
$E_{q}\left[f_{k}\right]$	Expected value of $f_{k}$ with respect to *q*
	Set of probability measures
$\lambda_{k}$	Lagrange multiplier associated to the constraint $E_{q}\left[f_{k}\right]={\langle f_{k}\rangle }_{e}$
H(x)	Energy function
*Z*	Partition Function
